# Myeloid NCOA4 sequesters KEAP1 to reduce ferroptosis for protection against salmonellosis in mice

**DOI:** 10.21203/rs.3.rs-4278310/v1

**Published:** 2024-05-16

**Authors:** Xiang Xue, Mariella Arcos, Zhaoli Liu, Luke Villareal, Paloma Kai Velez, Sharina Desai, Achraf Noureddine, David Martin, C Brinker, Donna Zhang

**Affiliations:** University of New Mexico; University of New Mexico; University of New Mexico; UNM; University of New Mexico; University of New Mexico; University of New Mexico; UNM; University of New Mexico; The Herbert Wertheim UF Scripps Institute for Biomedical Innovation and Technology

**Keywords:** Salmonella, macrophage, iron metabolism, oxidative stress

## Abstract

Salmonellosis is a severe infection caused by *Salmonella enterica* serovar Typhimurium, leading to significant global morbidity and mortality. Host nutrition immunity restricts extracellular bacterial replication by reducing iron availability through the induction of the antimicrobial hormone hepcidin. Hepcidin degrades the iron exporter ferroportin, limiting intestinal iron uptake and reducing iron transfer into the blood. Paradoxically, this defense mechanism increases iron storage in macrophages, fostering an environment conducive to intracellular pathogens like *Salmonella* to proliferate and disseminate. Mice lacking the iron storage protein ferritin heavy chain (FTH1) in myeloid cells exhibit worsened *Salmonella* infection. Nuclear receptor co-activator 4 (NCOA4) directs FTH1 autophagic degradation to release iron from storage during conditions of low iron. However, the role of myeloid NCOA4 in regulating salmonellosis and gut pathophysiology remains unclear. Here, we reveal that myeloid NCOA4 deficiency augments spleen iron levels and increases cellular iron accumulation, oxidative stress, and ferroptosis in bone marrow-derived macrophages (BMDM) cells. This deficiency also increases susceptibility to *Salmonella*-induced colitis in mice due to exacerbated oxidative stress and ferroptosis. Mechanistically, NCOA4 suppresses oxidative stress by directly binding to the E3 ubiquitin ligase Kelch-like ECH-associated protein 1 (KEAP1) and stabilizing the antioxidant transcription factor nuclear factor-erythroid 2-related factor 2 (NRF2). Activation of NRF2 protects myeloid NCOA4 knockout mice from *Salmonella*-induced colitis. Antioxidants Tempol and curcumin offer protection against colitis in myeloid NCOA4-deficient mice. A low iron diet and ferroptosis inhibition also mitigate the heightened colitis in these mice. Overexpression of myeloid cell-specific NCOA4 confers protection against *Salmonella*-induced colitis via upregulating NRF2 signaling. Together, this study not only advances our understanding of myeloid iron metabolism but also paves the way for novel myeloid cell-targeted therapies to combat salmonellosis.

## Introduction

Salmonellosis is an acute gastroenteritis, typically acquired orally through *Salmonella* enterica Serovar Typhimurium-contaminated water or food. Annually, an estimated 1.3 billion cases of Salmonella gastroenteritis occur, leading to approximately 3 million deaths worldwide (Kurtz et al., 2017). In the U.S., it leads to 1.35 million yearly illnesses, costing $2.8 billion (Wotzka et al., 2017). Interestingly, salmonellosis shows an association between high socioeconomic status and illness (Leon et al., 2015). Additionally, *Salmonella* is notorious as an enteric pathogen that infects a wide range of animal hosts, contributing to the deterioration of animal health, compromised animal welfare, enormous agricultural losses, and a significant medical burden worldwide (Tan et al., 2021). Thus, *Salmonella* infections pose a formidable threat to both public and animal health, incurring substantial annual costs.

Iron metabolism plays a vital role in host defense, as iron is essential for the growth of most bacteria. In response to infection-induced production of the proinflammatory cytokine interleukin 6, the antimicrobial hormone hepcidin, produced by the liver, orchestrates the endocytosis and degradation of the iron exporter ferroportin (Lokken et al., 2014). Consequently, dietary iron uptake and iron transfer into the blood are reduced, while macrophage iron storage in the liver and spleen is increased. This strategic process, known as nutritional immunity, aims to diminish the concentration of nutrients, including iron, in circulation, thereby depriving invading extracellular pathogens of essential resources (Núñez et al., 2018). Despite the host’s defense mechanisms, intracellular pathogens like *Salmonella* can exploit the host’s iron-withholding defense to thrive within host macrophage phagosomes and disseminate (Kim et al., 2014). Consequently, modulating host iron homeostasis presents an alternative approach to controlling multidrug-resistant intracellular bacteria.

Iron, playing an indispensable role in various cellular functions such as mitochondrial respiration and DNA synthesis (Berger et al., 2022), can also lead to the generation of reactive oxygen species (ROS), resulting in cellular damage and cell death (Dixon et al., 2014). To maintain cellular iron homeostasis, a finely tuned network of proteins is involved in iron import (transferrin, transferrin receptor [TFRC], and divalent metal transporter 1 [DMT1, encoded by SLC11A2 gene]), iron storage (ferritin), and iron export (ferroportin) (Morales and Xue, 2021). Hypoxia-inducible factor (HIF)-α plays a pivotal role in regulating several iron metabolic proteins, including TFRC, DMT1, and ferroportin, operating at the transcriptional level. Conversely, iron regulatory proteins (IRP) 1 and 2 modulate the protein levels of these iron metabolic proteins through post-transcriptional mechanisms. Over the past decade, our research has rigorously examined many of these iron metabolic proteins in pre-clinical models of gastrointestinal diseases (Xue et al., 2012, Xue et al., 2016, Xue et al., 2017, Schwartz et al., 2021, Kim et al., 2023). Recent findings from our research demonstrate the essential role of myeloid ferritin heavy chain (FTH1) in colitis and colitis-associated colorectal cancer under conditions of excess iron (Liu et al., 2023). Furthermore, colitis is reduced in mice treated with the Food and Drug Administration (FDA)-approved iron chelator deferiprone (DFP) or the superoxide dismutase mimetic antioxidant Tempol (Xue et al., 2017). Thus, targeting iron metabolism opens a new avenue for colitis treatment.

Similarly, targeting macrophage iron metabolism is central for controlling infections with intracellular pathogens like *Salmonella*. A recent report highlights the contribution of reduced hepcidin levels in children with severe malarial anemia to *Salmonella* growth, suggesting the iron availability limits bacterial proliferation (Abuga et al., 2021). Additionally, macrophage IRP1 and IRP2 play a pivotal role in restraining *Salmonella*. They induce lipocalin-2, a host antimicrobial factor that inhibits bacterial uptake of iron-laden siderophores, while simultaneously suppressing the ferritin iron pool (Nairz et al., 2015). Intriguingly, DMT1-deficient macrophages exhibit impaired control of *Salmonella* infection, characterized by increased intracellular iron due to reduced expression of ferroportin, accompanied by decreased levels of lipocalin-2 (Grander et al., 2022). Furthermore, mice lacking macrophage FTH1 show increased susceptibility to *Salmonella* infection, highlighting impaired cellular iron management, especially under iron overload (Haschka et al., 2021). NCOA4, a selective cargo receptor critical for intracellular iron homeostasis, facilitates ferritin iron storage or release in response to iron demand (Mancias et al., 2014). Ferritinophagy, the NCOA4 mediated autophagic degradation of ferritin, is essential for iron-dependent processes like erythropoiesis and mitochondrial heme synthesis (Santana-Codina et al., 2019; Ryu et al., 2017). Additionally, ferritinophagy regulates ferroptosis (Yu et al, 2022), a form of iron-dependent cell death associated with various diseases (Dixon et al., 2012, Ryan, 2023; Han et al., 2022; Mao et al., 2021). For example, ferritinophagy pumps iron into mitochondria, promoting cell survival and therapy resistance in pancreatic cancer (Jain et al., 2022; Ravichandran et al., 2022; Santana-Codina et al., 2022). In the context of liver specific NCOA4 modulation, knockdown increases while overexpression reduces mouse liver iron levels (Li et al., 2020; Li et al., 2022). Notably, NCOA4 whole-body knockout (KO) mice exhibit reduced systemic iron levels but increased liver and spleen iron levels, suggesting a tissue-specific effect (Bellelli et al., 2016). While myeloid NCOA4 deficiency expedites the clearance of *Mycobacterium* tuberculosis infection (Dai et al., 2023), further research is warranted to uncover its specific contribution to iron homeostasis and salmonellosis.

In this study, we revealed a non-canonical function of NCOA4, involving the suppression of oxidative stress during *Salmonella*-induced colitis. Myeloid NCOA4 deficiency has severe consequences, exacerbating salmonellosis by increasing oxidative stress and ferroptosis. Mechanistically, NCOA4 binds to KEAP1 and undergoes degradation through the autolysosomal pathway in wild-type cells. Consistently, myeloid NCOA4 overexpression protects mice from *Salmonella*-induced colitis via upregulating NRF2 signaling. This revelation represents the first indication that myeloid NCOA4 could serve as a promising therapeutic avenue for mitigating *Salmonella*-induced colitis.

## Results

### Myeloid NCOA4 deficiency augments spleen iron levels.

Prior research has demonstrated that whole-body NCOA4 KO mice exhibit decreased systemic iron but increased iron levels in specific tissues, including the spleen (Bellelli et al., 2016). Notably, myeloid cells, particularly monocytes and macrophages, contribute to spleen function by clearing aged or damaged red blood cells in the red pulp (Lewis et al, 2019). Under certain conditions like infections, alterations in myeloid cell activity in the spleen can impact overall immune system function and blood homeostasis (Bronte and Pittet, 2013). Our data reveals reduced mRNA levels of *Ncoa4* (**Fig. S1A**), but not the macrophage marker adhesion G protein-coupled receptor E1 (*Adgre1*, encoding the F4/80 protein, **Fig. S1B**) or the neutrophil marker *Ly6g* (**Fig. S1C**), in spleen tissues of KO mice compared to wildtype (WT, *Ncoa4*^F/F^) mice. Interestingly, the mRNA level of the *Tfrc* (**Fig. S1D**) was increased in the spleen tissues of KO mice compared to WT mice. Perl’s iron staining further confirms elevated iron levels in the splenic red pulp from KO mice compared to WT mice (**Fig. S1E, S1F**). This evidence underscores the crucial role of myeloid NCOA4 in regulating splenic iron levels.

### Myeloid NCOA4 depletion increases cellular iron, oxidative stress and ferroptosis levels in BMDM cells.

**Figure 1:**
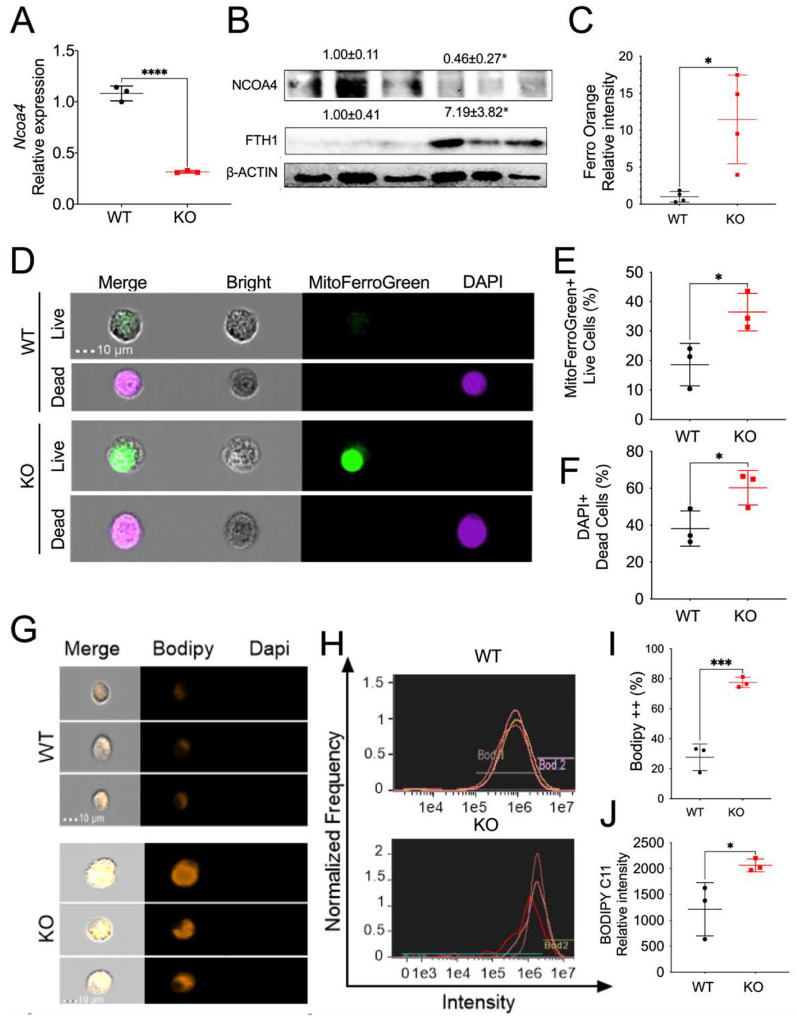
Myeloid NCOA4 depletion increases cellular iron, mitochondrial iron, cell death, and lipid peroxidation levels in BMDM cells. (**A**) qPCR analysis and (**B**) Immunoblot analysis, (**C**) Quantification of FerroOrange staining for cellular iron, (**D**) Representative imaging flow cytometry single cell images, (**E**) Quantification of mitoFerroGreen positive cells, (**F**) Quantification of DAPI positive dead cells, (**G**) Representative single cell images, (**H**) Histogram of imaging flow cytometry analysis of BODIPY C11 staining, (**I**) Positive BODIPY C11 staining cell percentage and (**J**) relative intensity quantification in BMDM from myeloid NCOA4 KO and WT mice (n=3–4). Values above blots represent mean ± S.D. Statistical significance was denoted as *p<0.05, ***p<0.001, ****p<0.0001 using Student’s t-test.

Two prominent features of ferroptosis are elevated iron levels and heightened intracellular oxidative stress, both of which are regulated by NCOA4 (Li K et al, 2022). Therefore, we explored the impact of myeloid NCOA4 KO on intracellular iron, oxidative stress, and cell death in BMDM. To further verify NCOA4 deletion in myeloid cells, we initially analyzed the mRNA and protein levels of NCOA4 in BMDM of KO and WT mice. qPCR analysis demonstrated that the mRNA expression of *Ncoa4* was significantly decreased (Fig. 1A). Immunoblot analysis revealed a significant decrease in the protein expression of NCOA4, along with an increase in FTH1, in BMDM from myeloid NCOA4 KO compared to their WT littermate control mice (Fig. 1B). This indicates impaired ferritinophagy function. Interestinlgy, FerroOrange staining indicated a notable rise in cellular iron levels (Fig. 1C), and MitoFerroGreen along with DAPI staining demonstrated increased mitochondrial iron and cell death in BMDM from myeloid NCOA4 KO mice (Fig. 1D–1F). Our qPCR analysis further demonstrated that the mRNA expression of iron uptake transporters *Slc11a2* and *Tfrc*, but not iron storage *Fth1* and iron exporter *Slc40a1*, were increased in BMDM cells from myeloid NCOA4 KO compared to their WT littermate control mice (**Fig. S2A-S2D**). These results support the concept that iron uptake mechanisms are upregulated in NCOA4 KO mice.

Our results also showed that myeloid NCOA4 KO mice exhibit elevated lipid peroxidation levels indicated by BODIPY C11 staining in BMDM (Fig. 1G–1J). However, our qPCR analysis demonstrated that the mRNA expression of antioxidant genes, including *Nrf2, Nqo1*, and *Ho-1*, was not changed in BMDM cells from myeloid NCOA4 KO mice compared to their WT littermate controls (**Fig. S2E-S2G**). Interestingly, the mRNA levels of the pro-inflammatory cytokine *Il1β*, but not *Tnfa* and *Il6*, were also increased (**Fig. S2H-S2J**). The mRNA levels of the pro-inflammatory M1 macrophage marker Nitric oxide synthase 2 (*Nos2*) (**Fig. S2K**) and the anti-inflammatory cytokine Interleukin 10 (*Il10*) (**Fig. S2L**) did not show a significant change. These data indicate a mild increase in ferroptosis in the BMDM from KO mice at the basal level.

### Myeloid NCOA4 deficiency in mice increases susceptibility to Salmonella-induced colitis.

**Figure 2:**
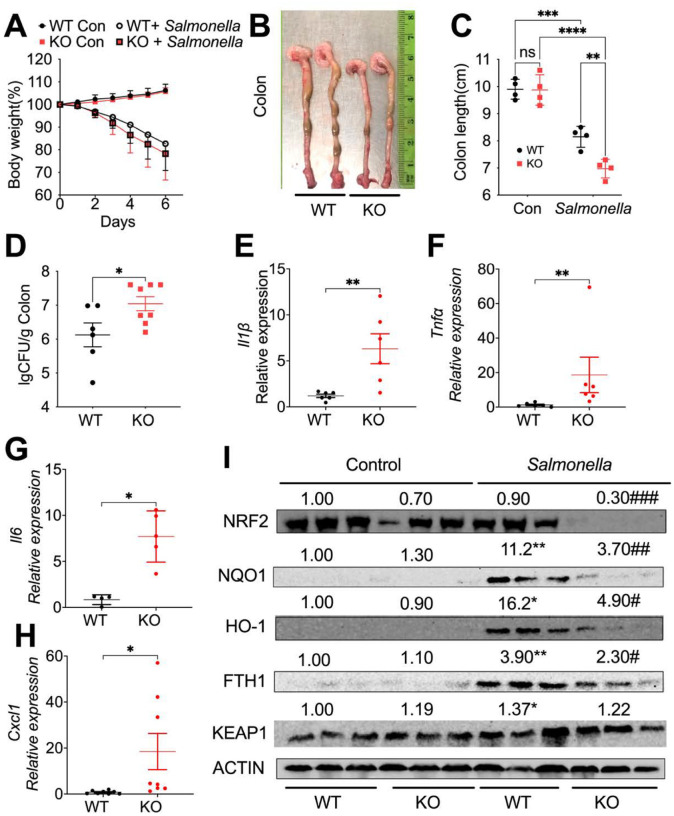
Myeloid NCOA4 knockout mice display shortened colon length, impaired *Salmonella* clearance, heightened inflammation, and reduced NRF2 signaling antioxidant proteins in *Salmonella*-treated mouse colon tissues. (**A**) Body weight, (**B**) representative images of dissected colon, (**C**) colon length, (**D**) Colony Formation Unit (CFU) of *Salmonella*, qPCR analysis of the proinflammatory cytokines (**E**) *Il1β*, (**F**) *Tnfa*, (**G**)*Il6*, and (**H**) *Cxcl1*, (**I**) immunoblot analysis, were conducted on colon tissues from control or *Salmonella*-treated KO mice (n=3–8) and WT mice (n=3–6). ns, not significant. *p<0.05, **p<0.01, ***p<0.001, ****p<0.0001 Vs WT. #p<0.05, ##p<0.01, ###p<0.001 Vs Salmonella*treated* WT mice. Two-way ANOVA followed by Tukey’s multiple comparisons test for (C and I). Student’s t-test for (D-H).

A recent publication demonstrated that myeloid FTH1 KO mice experience deteriorated cellular iron handling, exacerbating *Salmonella* infection by triggering hyperinflammation (Haschka et al., 2021). Thus, we conducted further studies on the role of myeloid NCOA4 in controlling infections with the intracellular pathogen *Salmonella*. Our preliminary findings indicate that myeloid NCOA4 KO mice, compared to WT, experience similar body weight loss (Fig. 2A) and histopathological changes (**Fig. S3A, S3B**) but demonstrate shortened colon length (Fig. 2B, 2C), impaired *Salmonella* clearance (Fig. 2D), and elevated expression of proinflammatory cytokines including *Il1β* (Fig. 2E), *Tnfa* (Fig. 2F), *Il6* (Fig. 2G) and *Cxcl1* (Fig. 2H). These results highlight a pivotal role for NCOA4 in not only controlling bacterial spread but also in regulating proinflammatory responses within the colon. Importantly, this function is distinct from its well-established role in ferritinophagy, underscoring the multifaceted nature of NCOA4 in the context of colonic homeostasis and immune regulation.

Our results further showed that *Salmonella* infection in WT mice activates NRF2 signaling, as evidenced by increased protein levels of its downstream target antioxidant proteins, including NQO1, HO-1, and FTH1, in the mouse colon tissues (Fig. 2I). Conversely, myeloid NCOA4 KO mice exhibit diminished NRF2 signaling molecules, including NRF2, NQO1, HO-1, and FTH1 (Fig. 2I), in *Salmonella*-treated mouse colon tissues. In WT mice, *Salmonella* increased levels of a key negative regulator of the NRF2 antioxidant signaling pathway KEAP1 (Fig. 2I). However, these increases were not observed in the KO mice. Consistently, the mRNA levels of *Nrf2* and *Nqo1* were significantly decreased in the *Salmonella*-treated colon tissues from the KO mice compared to WT mice (**Fig. S3C, S3D**). Analysis of colon tissues treated with *Salmonella* revealed increased mRNA levels of *Tfrc* (Fig. S3E), reduced mRNA levels of *Ncoa4* (**Fig. S3F**), and increased mRNA levels of *Nos2* (**Fig. S3G**), but no changes were observed in the mRNA levels of *Il10* (**Fig. S3H**). Furthermore, the antimicrobial peptides regenerating islet-derived 3 beta (*Reg3b*) (**Fig.S3I**) and calcium-binding protein A8 (*S100a8*) (**Fig.S3J**) but not the siderophore Lipocalin 2 (*Lcn2*) (**Fig.S3K**). These results suggest that the deficiency of myeloid NCOA4 enhances the suppression of the NRF2 signaling pathway, leading to increased oxidative stress in *Salmonella*-treated mouse colon tissues.

### NCOA4 suppresses oxidative stress by directly binding to KEAP1, thereby stabilizing NRF2.

**Figure 3:**
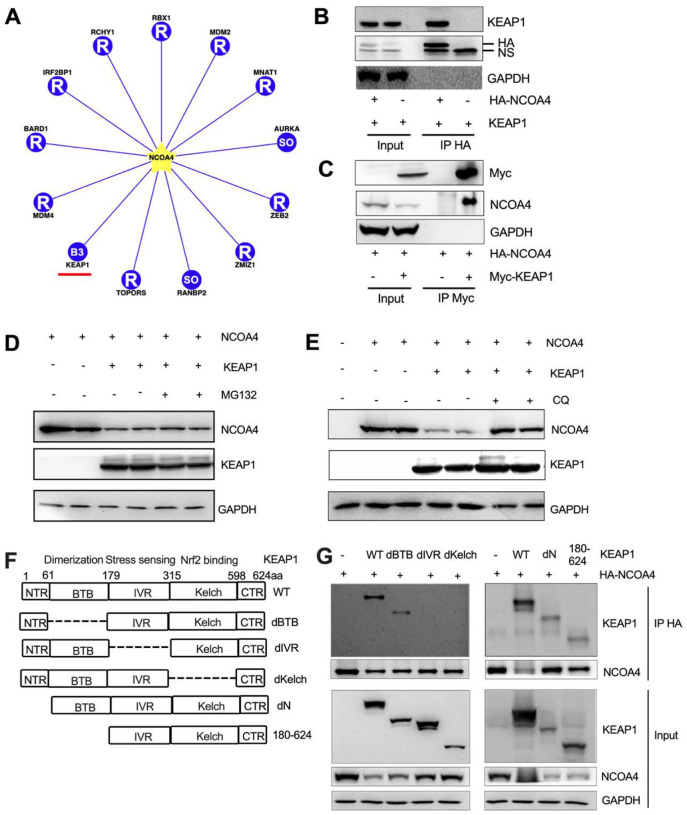
The IVR and Kelch domains of KEAP1 are essential for NCOA4 binding and its degradation through lysosome but not proteasome. (**A**) Predicted (13 high confidence) interaction of E3 (including KEAP1) and substrate (NCOA4) in Ubibrowser database. Co-immunoprecipitation (IP) analysis using (**B**) anti-HA or (**C**) anti-Myc antibody in HEK293 cells co-transfected with Myc-KEAP1 and HA-NCOA4 plasmids. Immunoblot analysis in HEK293T cellstransiently transfected with NCOA4 and KEAP1 plasmids with or without (**D**) a proteasomal inhibitor 10 μM MG132 treatment for 4h, (**E**) an autolysosomal inhibitor 150 μM Chloroquine (CQ) for 24h. (**F**) Diagram of truncated constructs of KEAP1. Each domain (NTR, BTB, IVR, Kelch and CTR) of the KEAP1 protein (indicated by boxes), its putative molecular function, and number of amino acids are shown. (**G**) Co-IP analysis in HEK293 cells co-transfected with different KEAP1 and HA-NCOA4 plasmids. To detect different KEAP1 mutant constructs, we used two KEAP1 antibodies with different immunogens: Left, Santa Cruz, sc-514914, 39–65aa. Right, Proteintech, 10503–2-AP, 325–624aa.

The molecular basis for the interaction between NCOA4 and NRF2 is still missing so far. Using Ubibrowser, a comprehensive database for proteome-wide known and predicted ubiquitin ligase/deubiquitinase-substrate interactions in eukaryotic species (Wang et al, 2022), we found that NCOA4 is a high confidence E3 ligase substrate of KEAP1 (Fig. 3A). Our reciprocal co-immunoprecipitation assay confirmed that NCOA4 directly interacts with KEAP1 (Fig. 3B, 3C). Furthermore, our data highlight a distinctive interaction between endogenous NCOA4 and KEAP1 in human cells (**Fig. S4A**), suggesting the physiological relevance of the KEAP1-NCOA4 interaction.

Under iron-replete conditions, NCOA4 binding by HERC2, an E3 ubiquitin ligase, is increased, leading to proteasomal degradation of NCOA4 (Mancias et al., 2015). Given that KEAP1 is also a E3 ubiquitin ligase, we hypothesized that it causes NCOA4 ubiquitination and proteasomal degradation. Indeed, KEAP1 overexpression reduced NCOA4 levels, but the reduced NCOA4 was not restored by the proteasomal inhibitor MG132 (Fig. 3D). Ectopic expression of KEAP1 decreases the expression of IKKβ via autophagic degradation but not proteasomal degradation (Kim et al, 2010). Moreover, treatment with the autophagic flux inhibitor Bafilomycin A1 stabilizes NCOA4 in H4 neuroglioma cells (Goodwin et al., 2017). Thus, we further tested the effect of autolysosomal inhibition via chloroquine on KEAP1 mediated NCOA4 degradation. Interestingly, the KEAP1 overexpression reduced NCOA4 was restored by chloroquine (Fig. 3E), suggesting that KEAP1 mediates autolysosomal degradation of NCOA4.

KEAP1 is a multidomain homodimeric protein which has five distinct domains (Fig. 3F, Dinkova-Kostova et al, 2012, Shibata et al, 2008): (i) NTR: N-terminal region (amino acids 1 – 60); (ii) BTB: broad complex, Tramtrack, Bric- ábrac (amino acids 61 – 179)–the domain through which KEAP1 dimerizes; (iii) IVR: intervening region (amino acids 180–314) which is a particularly cysteine-rich region containing 8 cysteine residues among its 134 amino acids; (iv) Kelch domain (amino acids 315–598)–the domain through which KEAP1 binds to NRF2; and (v) CTR: C terminal region (amino acids 599 – 624). To determine the exact NCOA4 binding motifs in the KEAP1 protein, we have co-transfected different KEAP1 mutant plasmids (deltaN, deltaBTB, deltaIVR, deltaKelch and 180–625 aa, Lau et al, 2010) with HA-NCOA4. We found that the IVR and Kelch domains of KEAP1 protein are essential for NCOA4 binding (Fig. 3G). Furthermore, KEAP1 overexpression reduced the protein expression levels of NRF2, whereas co-transfection with NCOA4 partially restored the levels of NRF2 (**Figure S4B**). These results support our hypothesis that NCOA4 and NRF2 are competing for the same KEAP1 binding domain.

### Activation of NRF2 protects myeloid NCOA4 KO mice from Salmonella-induced colitis.

**Figure 4:**
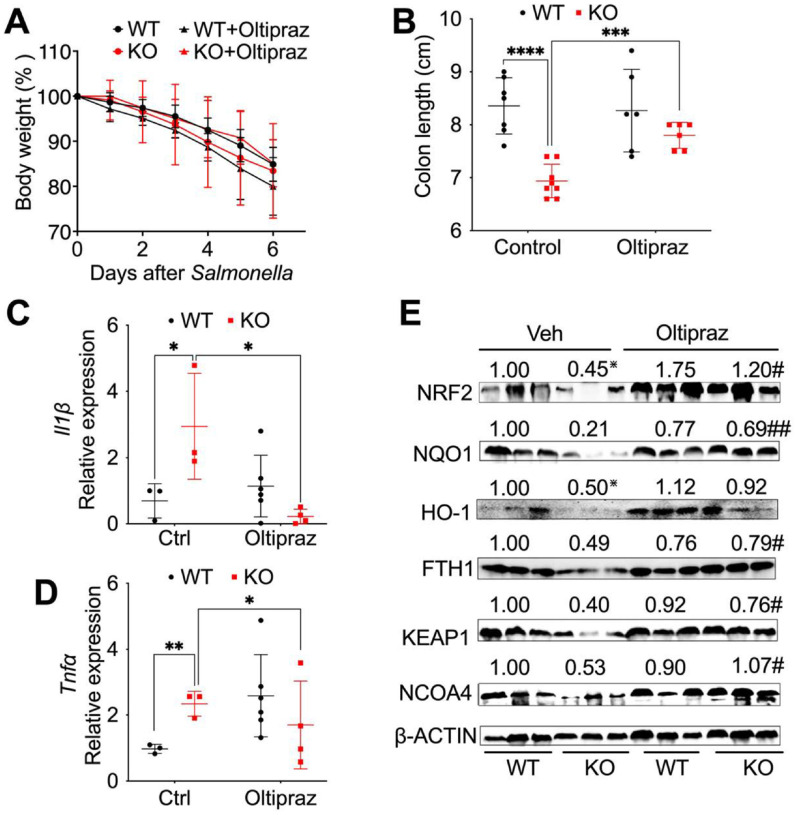
Activation of NRF2 protects myeloid NCOA4 KO mice from *Salmonella*-induced colitis. (**A**) Body weights and (**B**) colon lengths, qPCR analysis of the proinflammatory cytokines (**C**) *Il1β* and (**D**) *Tnfa*, (**E**) Immunoblot analysis of key antioxidant proteins in the NRF2 signaling pathway and NCOA4 were conducted on colon tissues from control or *Salmonella*-treated KO mice (n=3–8) and WT mice (n=3–7) treated with either vehicle control or NRF2 activator Oltipraz. Statistical significance was denoted as *p<0.05, **p<0.01, ***p<0.001, ****p<0.0001 vs WT control or #p<0.05, ##p<0.01 vs KO control. Two-way ANOVA followed by Tukey’s multiple comparisons test was applied.

To further assess the role of NRF2 signaling in NCOA4 deficiency-enhanced colitis, NCOA4 KO and WT mice were induced with colitis using *Salmonella*. Subsequently, mice were treated with either vehicle or daily oral gavage of 500 mg/kg NRF2 activator Oltipraz (Ramos-Gomez et al, 2001) through intraperitoneal injection (Kim et al, 2019; Silva et al, 2022). Activating NRF2 didn’t influence mouse body weight loss (Fig. 4A) but significantly increased the colon lengths in myeloid NCOA4 KO mice (Fig. 4B). Furthermore, NRF2 activation significantly decreased pro-inflammatory cytokines including *Il1β* (Fig. 4C) and *Tnfa* (Fig. 4D), indicating protective effects in myeloid NCOA4 KO mice. In addition, activation of NRF2 resulted in the restoration of NRF2 signaling proteins (NRF2, NQO1, FTH1 and KEAP1) as well as NCOA4 in colon tissues obtained from myeloid NCOA4 KO mice treated with *Salmonella* (Fig. 4E). This suggests an on-target drug effect and highlights a competitive relationship between NRF2 and NCOA4.

### Antioxidants Tempol and curcumin protect against Salmonella-induced colitis in mice lacking myeloid NCOA4.

**Figure 5:**
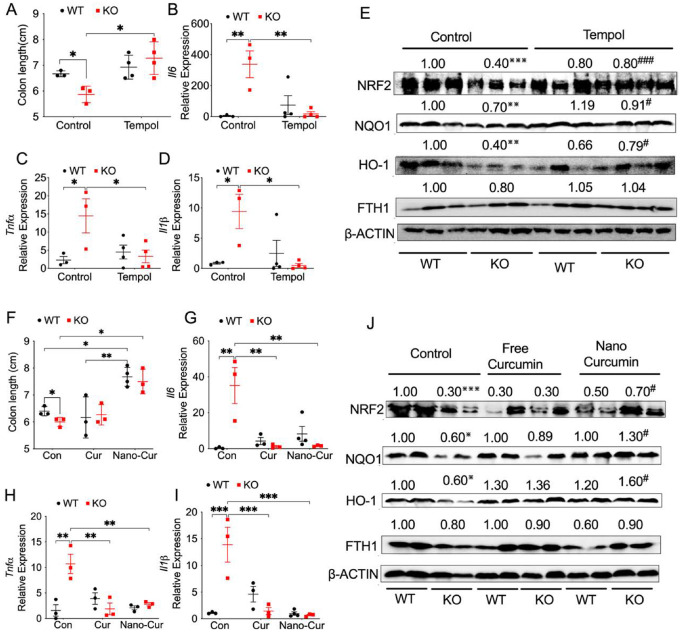
Antioxidants Tempol and Myeloid Cell Targeting Nanoparticles Conjugated Curcumin Protect Against *Salmonella*-Induced Colitis in Myeloid NCOA4-Deficient Mice. (**A**) Colon length, qPCR analysis of the proinflammatory cytokines (**B**) *Il6*,, (**C**) *Tnfa*, and (**D**) *Il1β*, (**E**) immunoblot analysis of key antioxidant proteins in the NRF2 signaling pathway in colon tissues from *Salmonella*-treated KO mice (n=3–4) and WT mice (n=3–4) that received vehicle or 0.064% Tempol in drinking water. (**F**) Colon length, qPCR analysis of the proinflammatory cytokines (**G**) *Il6*, (**H**) *Tnfa*, and (**I**) *Il1β*, (**J**) immunoblot analysis of key antioxidant proteins in the NRF2 signaling pathway in colon tissues from *Salmonella*-treated KO mice (n=3–4) and WT mice (n=3–4) that received curcumin (Cur) or myeloid cell targeting nanoparticles conjugated curcumin (nano-Cur) treatment. Statistical significance was denoted as *p<0.05, **p<0.01, ***p<0.001 or #p<0.05, ###p<0.001 vs KO control. Two-way ANOVA followed by Tukey’s multiple comparisons test was applied.

We have demonstrated that dextran sulfate sodium-induced acute colitis is diminished in mice treated with Tempol (Xue et al., 2017). Here we conducted further investigations into the role of Tempol in controlling *Salmonella*-induced colitis. Tempol didn’t influence mouse body weight loss (**Fig. S5A**). However, Tempol significantly protected myeloid NCOA4 KO mice but not WT mice with *Salmonella*-induced colitis, as evidenced by increased colon length (Fig. 5A) and reduced levels of proinflammatory cytokines including *Il1β* (Fig. 5B), *Tnfa* (Fig. 5C) and *Il6* (Fig. 5D). In addition, Tempol also led to the restoration of NRF2 signaling proteins (NRF2, NQO1 and HO-1) in colon tissues obtained from myeloid NCOA4 KO mice treated with *Salmonella* (Fig. 5E). Interestingly, the protein levels of FTH1 were not significantly changed (Fig. 5E). This suggests Tempol can reduce oxidative stress and alleviate colitis caused by *Salmonella* infection in mice lacking myeloid NCOA4.

Considering the synthetic nature of Tempol and aiming for future clinical therapeutic and prophylactic applications with enhanced safety, we investigated the potential of using the naturally occurring antioxidant curcumin. Curcumin (C_21_H_2_OO_6_) is a lipophilic substance of a polyphenol nature, obtained from rhizomes of turmeric (*Curcuma longa* L.). It has antibacterial and antioxidant properties, and because it is a natural compound and low cost, curcumin exhibits great promise as a therapeutic agent to develop new treatments for bacterial infections and oxidative stress-related disease. However, the role of curcumin in controlling *Salmonella* is a subject of controversy (Leyva-Diaz et al, 2021). Previous publications have demonstrated the antioxidant effect of curcumin (Jakubczyk et al., 2020). However, one report suggests that curcumin can enhance the pathogenicity of *Salmonella* by increasing its robustness, possibly through the upregulation of genes involved in resistance against microbial peptides (Marathe et al., 2010). We hypothesize that specific delivery of curcumin into myeloid cells might mitigate potential side effects. First, we performed *in vitro* studies with RAW264.7 cells stimulated with lipopolysaccharide (LPS), an inflammatory stimulant, to compare the effects of free curcumin and nanoparticles conjugated with curcumin (nano-curcumin). Our results showed an increase in the protein levels of NCOA4 following stimulation with LPS concentrations higher than 1μg/mL (**Fig. S5B**). While both free and nano-curcumin were not able to reduce the mRNA expression levels of *Tnfa* in LPS challenged RAW264.7 cells (**Fig. S5C**), nano-curcumin have a slightly stronger effect on inhibiting LPS increased *Il1β* mRNA levels (**Fig. S5D**). Next, we treated mice with *salmonella* and the next day with free or nano-curcumin through intraperitoneal injection. Myeloid cell-targeting nano-curcumin didn’t have a significant improvement on the body weight (**Fig. S5E**). However, nano-curcumin provided significantly better protection in mice with *Salmonella*-induced colitis, as indicated by increased colon length (Fig. 5F). Interestingly, both curcumin and nano-curcumin similarly reduced the levels of proinflammatory cytokines in myeloid NCOA4 KO but not WT mice (Fig. 5G–5I). Additionally, nano-curcumin also led to the restoration in NRF2 signaling proteins (NRF2, NQO1, and HO-1) in colon tissues from myeloid NCOA4 KO mice (Fig. 5J). Consistently, the protein levels of FTH1 were not significantly changed (Fig. 5J). This suggests nano-curcumin can reduce oxidative stress and alleviate colitis caused by *Salmonella* infection in mice lacking myeloid NCOA4.

Together, antioxidants may alleviate colitis severity in myeloid NCOA4-deficient individuals but could impede macrophages’ ability to combat *Salmonella* in WT mice due to the direct antimicrobial functions of ROS (Herb and Schramm, 2021). This underscores the need for a nuanced approach in considering antioxidant therapy based on the specific immune context.

### A low iron diet and ferroptosis inhibition protect mice from Salmonella-induced colitis enhanced by myeloid NCOA4 depletion.

**Figure 6:**
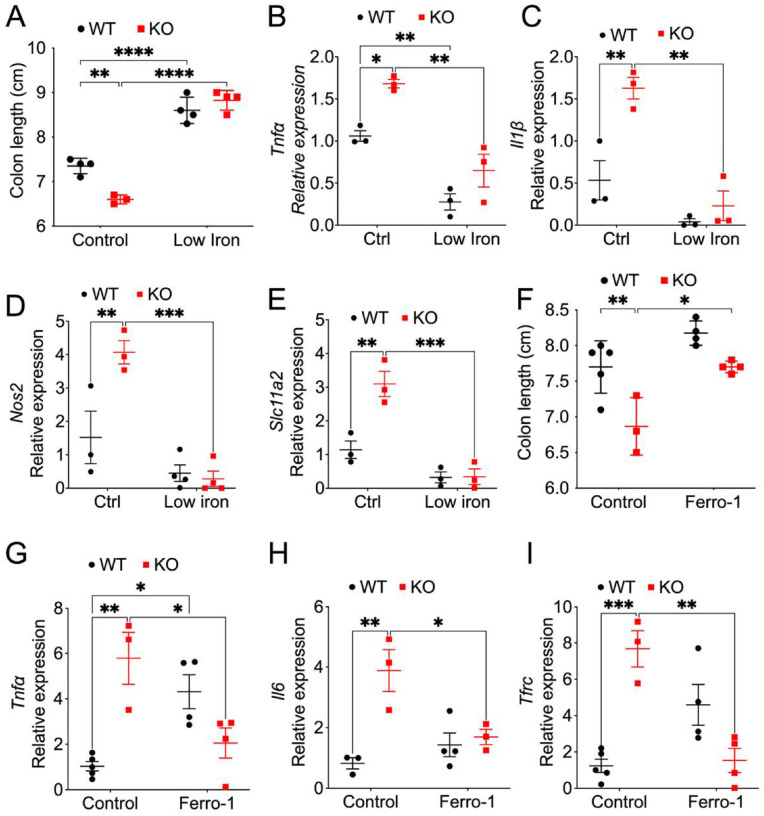
A Low Iron Diet and ferroptosis inhibition abolishes the susceptibility of myeloid NCOA4 knockout mice to *Salmonella*-induced colitis. (**A**) colon length, qPCR analysis of the proinflammatory cytokines (**B**) *Tnfa* and (**C**) *Il1β*, (**D**)*Nos2* and (**E**) S*ic11a2* in colon tissues from *Salmonella*-treated KO mice (n=3–4) and WT mice (n=3–4) that received either a control (40 ppm iron) or a low iron (3.5 ppm iron) diet. (**F**) Colon length, qPCR analysis of proinflammatory cytokines (**G**) *Tnfa* and (**H**) *Il6*, (**I**) iron uptake transporter *Tfrc* in colon tissues from myeloid NCOA4 KO and their WT control mice treated with vehicle control (n=3–5) or a daily 1 mg/kg dose of Ferrostatin-1 (n=3–4) via i.p. injection starting 2 days before the *Salmonella* infection. Statistical significance was denoted as *p<0.05, **p<0.01, ***p<0.001, ****p<0.0001. Two-way ANOVA followed by Tukey’s multiple comparisons test was applied.

*Salmonella* relies on iron for growth in monocyte-macrophage system cells (Nairz, et al, 2014). A low iron diet didn’t have a significant improvement on the body weight (Fig. S6A). However, the protective impact of the low-iron diet is evident in increased colon length (Fig. 6A) and decreased levels of proinflammatory cytokines including *Tnfa* (Fig. 6B), *Il1β* (Fig. 6C) and *Nos2* (Fig. 6D) in both myeloid NCOA4 KO and WT mice. Additionally, the low iron diet effectively decreased the mRNA levels of *Slc11a2* (Fig. 6E). These findings emphasize the crucial role of dietary iron levels in influencing colitis severity, proposing a potential avenue for modulating colitis outcomes through dietary interventions that target iron availability.

Recent research has demonstrated that ferroptosis sensitivity is modulated by NCOA4, given the central role of NCOA4-mediated ferritinophagy in regulating intracellular iron levels (Santana-Codina et al., 2018). A ferroptosis inhibitor Ferrostatin-1 didn’t have a significant improvement on the body weight (**Fig. S6B**). However, our data reveal that colon length was reduced in myeloid NCOA4 KO mice compared to WT mice 7 days after *Salmonella* infection, an effect rescued by Ferrostatin-1 (Fig. 6F). Similarly, proinflammatory cytokine *Tnfa* (Fig. 6G), *Il6* (Fig. 6H) and iron uptake transporter *Tfrc* (Fig. 6I) were elevated in colons from myeloid NCOA4 KO mice compared to WT mice 7 days after *Salmonella* infection, with ferroptosis inhibitor Ferrostatin-1 also rescuing these effects. Thus, these data suggest that the heightened susceptibility to *Salmonella*-induced colitis in myeloid NCOA4 knockout mice is attributable to ferroptosis.

### Myeloid cell-specific NCOA4 overexpression protects mice from Salmonella-induced colitis.

**Figure 7:**
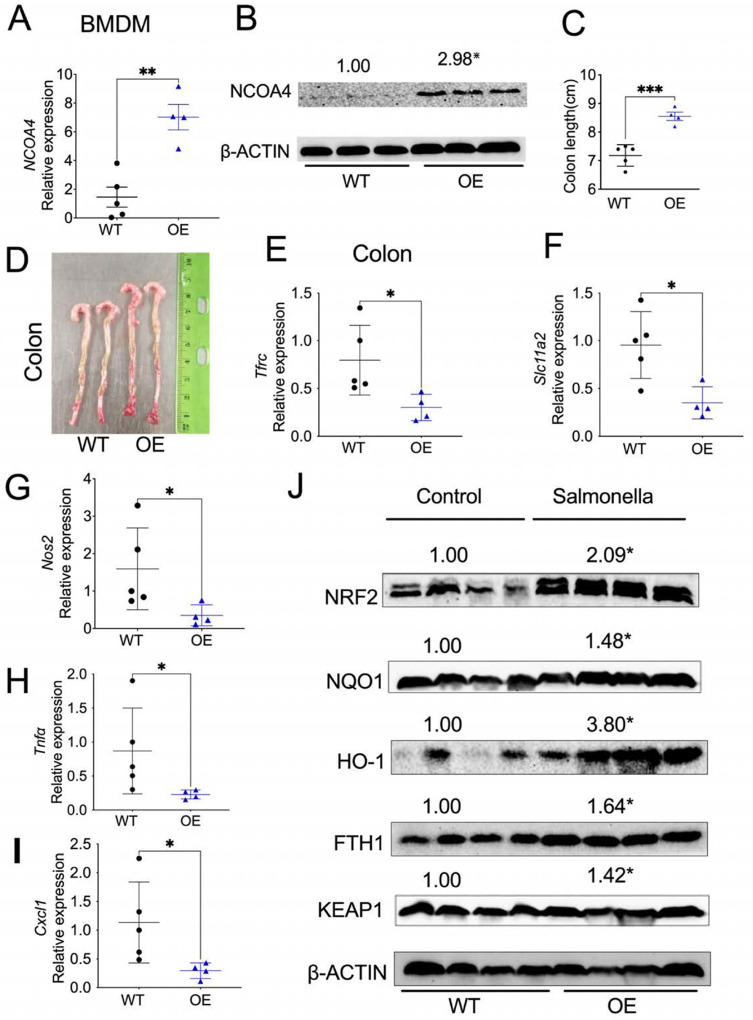
Myeloid cell-specific NCOA4 overexpression protects mice from *Salmonella*-induced colitis. (**A**) qPCR analysis and (**B**) immunoblot analysis of *NCOA4* in BMDM cells from WT (n=3–4) and myeloid cell-specific NCOA4 OE (n=3–4) mice. (**C**) colon lengths and (**D**) representative image of colons, and qPCR analysis of (**E**) *Tfrc*, (**F**) *Slc11a2*, (**G**) *Nos2* and (**H**) *Tnfa*, (**I**) *Cxcl1* in colons from OE (n=4) and WT mice (n=4–5). *p<0.05, **p<0.01, ***p<0.001, Student’s t-test.

To assess if myeloid NCOA4 is sufficient to protect mice from *Salmonella*-induced colitis, we generated myeloid cell specific NCOA4 OE mice. qPCR analysis confirmed that NCOA4 is increased in the spleens of OE mice compared to WT mice (**Fig. S7A**). Also, qPCR and immunoblot analysis confirmed that NCOA4 mRNA (Fig. 7A) and protein (Fig. 7B) expression levels are significantly increased in BMDM cells from OE mice than WT mice. Interestingly, we observed increased mRNA expression of *Nrf2* (**Fig. S7B**), *Nqo1* (**Fig. S7C**), and *Hmox1* (**Fig. S7D**) in BMDM cells from OE mice compared to WT mice. However, there was no significant change in the pro-inflammatory cytokine *Il1β* (**Fig. S7E**).

Although there is no significant change in body weight (**Fig. S7F**), the OE mice exhibit protection from *Salmonella*-induced colitis, as indicated by longer colon lengths (Fig. 7C, 7D). Interestingly, analysis of colon tissues treated with *Salmonella* showed decreased expression of *Tfrc* (Fig. 7E), *Slc11a2* (Fig. 7F), *Nos2*(Fig. 7G), *Tnfa* (Fig. 7H) and *Cxcl1* (Fig. 7I), confirming that NCOA4 OE diminishes inflammation due to *Salmonella* infection by downregulating iron levels. Further analysis confirmed increased protein levels of NRF2, NQO1, HO-1, FTH-1 and KEAP1 in these colon tissues (Fig. 7J). These results demonstrate that NCOA4 OE manifests less severe colitis and oxidative stress.

## Discussion

Traditionally, researchers have primarily viewed NCOA4 as engaged in ferritinophagy (Mancias et al., 2014) or as an androgen receptor coactivator (Yeh et al., 1996). However, our groundbreaking research challenges this perception by unveiling a non-canonical role of NCOA4. Specifically, we have discovered that NCOA4 suppresses oxidative stress by directly binding to KEAP1, thereby stabilizing NRF2 and mitigating ferroptosis. This revelation represents the first indication that myeloid NCOA4 could serve as a promising therapeutic avenue for mitigating *Salmonella*-induced colitis.

Interestingly, infection of myeloid NCOA4-deficient mice with *Salmonella* resulted in worsened inflammation and bacterial load compared to WT mice. Notably, another study involving a different intracellular bacterium, *Mycobacterium* tuberculosis, demonstrated that NCOA4 deficiency in myeloid cells expedites the clearance of *Mycobacterium* (Dai et al., 2023). Although both bacteria reside intracellularly, a crucial difference lies in their exact localization: while *Mycobacterium* resides in the early phagosome, *Salmonella* primarily inhabits the late phagosome (Haschka et al., 2021). This distinction in intracellular localization emphasizes the importance of considering the precise localization of intracellular pathogens.

Our findings reveal novel mechanisms rooted in dysfunction within myeloid iron metabolism, offering a deeper understanding of the intricate interplay between iron metabolism and oxidative stress. Notably, the NCOA4 protein lacks the canonical KEAP1 binding motifs found in other proteins such as NRF2 (ETGE and DLG, Tong et al., 2006) and p62 (DPSTGE, Lau et al., 2010). Instead, NCOA4 comprises the N-terminal coiled-coil region (NTR), middle domain (M), and C-terminal region (CTR), interconnected with intrinsically disordered regions (IDRs; IDR1 and IDR2, Ohshima et al., 2022). Further investigation will be necessary to determine which region of NCOA4 is critical for its interaction with KEAP1.

Conceptually, our work pioneers the understanding of how NCOA4 influences oxidative stress, leading to the reprogramming of cellular iron metabolism and identifying it as a protective mechanism in colitis. This conceptual innovation lays the groundwork for unlocking targeted therapeutic avenues and mitigating unintended side effects. Our results demonstrate that dietary iron has a protective impact in myeloid NCOA4 KO mice, but reducing iron intake from the diet can lead to secondary effects such as anemia. Finding a method to reduce iron levels without causing side effects is ideal, and our research suggests that nanoparticles may offer a more efficient solution. Therefore, investigating whether iron chelator-embedded nanoparticles exhibit protective effects against *Salmonella* infection would be an intriguing avenue for future research.

In conclusion, the strength and innovation of this work are underscored by our pioneering exploration of how micronutrients intricately shape mucosal immunity. This approach illuminates the pivotal role of myeloid cells in the narrative of salmonellosis, promising a leap forward in precision cell targeting as we unravel the enigma of micronutrient metabolism for a more nuanced approach to salmonellosis treatment.

## Data Availability

For original data, please contact xxue@salud.unm.edu.
